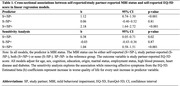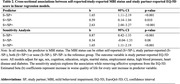# A CAN‐PROTECT study: The effect of self‐reported and study partner‐reported mild behavioral impairment on quality of life in older adults without dementia

**DOI:** 10.1002/alz.090348

**Published:** 2025-01-03

**Authors:** Ibadat Warring, Dylan X. Guan, Zahinoor Ismail, Eric E. Smith

**Affiliations:** ^1^ University of Calgary, Calgary, AB Canada; ^2^ Hotchkiss Brain Institute, University of Calgary, Calgary, AB Canada; ^3^ Department of Clinical Neurosciences and Hotchkiss Brain Institute, University of Calgary, Calgary, AB Canada

## Abstract

**Background:**

Mild behavioral impairment (MBI) is a syndrome that leverages neuropsychiatric symptoms that emerge in later‐life, and which persist, to identify individuals at high‐risk for incident dementia. Attendant with MBI are changes in quality of life (QoL), which can present concurrent with the onset of cognitive decline or even before. Obtaining information from participants and study partners can provide a broader overview of health and QoL. We investigated the relationship between MBI and QoL in dementia‐free older adults, incorporating both self‐ and study partner reports.

**Methods:**

Data (n = 608) are from the CAN‐PROTECT study. MBI status (±) was determined with a MBI Checklist cut‐off score ≥8 based on self‐ or study‐partner report, resulting in a four‐level categorical variable of MBI positivity/negativity (S‐/SP‐[concordant MBI‐], S+/SP‐[discordant MBI S+], S‐/SP+[discordant MBI SP+], S+/SP+[concordant MBI+]). QoL was assessed using the self‐ and study‐partner‐reported EQ‐5D (higher scores reflecting poorer QoL). Linear regression modelled associations between MBI status (exposure) and EQ‐5D total score (outcome), adjusted for age, cognition, sex, education, ethnocultural origin, marital status, employment status, high blood pressure, diabetes, and heart disease. A sensitivity analysis removed EQ‐5D affective symptoms.

**Results:**

Participants (mean age = 64.8±7.1, 77.0% female) had mean self‐reported scores of 4.6±6.7 for MBI and 6.7±1.8 for EQ‐5D. Study partners reported mean scores of 3.7±5.8 for MBI and 1.4±0.4 for EQ‐5D. Distribution of MBI status was 71.9% for concordant MBI‐, 13.3% for discordant S+, 8.3% for discordant SP+ and 6.6% for concordant MBI+. QoL was poorer in the concordant MBI+ group for self‐reported (b = 2.48, 95% confidence interval [95%CI]: 1.96–3.00, p<.001) (Table 1) and study‐partner reported EQ‐5D (b = 2.63, 95%CI: 2.00‐3.27, p<.001) (Table 2), compared to concordant MBI‐ group. There was a significant association with discordant MBI S+ (p<.001) but not discordant MBI SP+ (p<.81) with self‐reported EQ‐5D. Both discordant groups were significant with study‐partner reported EQ‐5D.

**Conclusion:**

In a mostly cognitively unimpaired community dwelling sample, MBI associations with self‐ and study‐partner‐reported QoL were strongest when MBI symptoms were endorsed by both participants and their study partners. These findings underscore the importance of including study partners brain aging research across the cognitive continuum.